# Uncovering of intraspecies macular heterogeneity in cynomolgus monkeys using hybrid machine learning optical coherence tomography image segmentation

**DOI:** 10.1038/s41598-021-99704-z

**Published:** 2021-10-19

**Authors:** Peter M. Maloca, Christine Seeger, Helen Booler, Philippe Valmaggia, Ken Kawamoto, Qayim Kaba, Nadja Inglin, Konstantinos Balaskas, Catherine Egan, Adnan Tufail, Hendrik P. N. Scholl, Pascal W. Hasler, Nora Denk

**Affiliations:** 1 Department of Ophthalmology, University of Basel, 4031 Basel, Switzerland; 2 Institute of Molecular and Clinical Ophthalmology Basel (IOB), 4031 Basel, Switzerland; 3 Moorfields Eye Hospital NHS Foundation Trust, London, EC1V 2PD UK; 4 Preclinical Research and Early Development, Pharmaceutical Sciences, Hoffmann-La Roche, 4070 Basel, Switzerland; 5 Moorfields Ophthalmic Reading Centre, London, UK

**Keywords:** Translational research, Medical research, Experimental models of disease

## Abstract

The fovea is a depression in the center of the macula and is the site of the highest visual acuity. Optical coherence tomography (OCT) has contributed considerably in elucidating the pathologic changes in the fovea and is now being considered as an accompanying imaging method in drug development, such as antivascular endothelial growth factor and its safety profiling. Because animal numbers are limited in preclinical studies and automatized image evaluation tools have not yet been routinely employed, essential reference data describing the morphologic variations in macular thickness in laboratory cynomolgus monkeys are sparse to nonexistent. A hybrid machine learning algorithm was applied for automated OCT image processing and measurements of central retina thickness and surface area values. Morphological variations and the effects of sex and geographical origin were determined. Based on our findings, the fovea parameters are specific to the geographic origin. Despite morphological similarities among cynomolgus monkeys, considerable variations in the foveolar contour, even within the same species but from different geographic origins, were found. The results of the reference database show that not only the entire retinal thickness, but also the macular subfields, should be considered when designing preclinical studies and in the interpretation of foveal data.

## Introduction

The importance of nonhuman primates as models for a multitude of human diseases has been well documented^1–3^. Particularly, research in cynomolgus macaques (*Macaca fascicularis*) has provided essential insights for the development, nonclinical ocular safety profiling, and therapeutic interventions of drugs, especially for agents or gene therapy administered in the fovea^4–9^. The fovea represents a depression in the middle of the macula and is the site of the highest cone concentration, which is designated as the central bouquet of cones^10^.

The entire macular thickness represents an important biomarker in assessing a large number of retinal pathologies in humans and nonhuman primates^11,12^. For example, changes in macular thickness were examined in cynomolgus monkeys for the safety assessment of retinal therapies, such as antivascular endothelial growth factor (anti-VEGF) administration^13–15^. These studies have paved the way for a clinical application in humans and established the use of anti-VEGF therapy as a reliable treatment of neovascular age-related macular degeneration^16,17^, representing, to date, one of the most frequent and successful ocular interventions^18^. In this context, the use of optical coherence tomography (OCT) as a noninvasive, longitudinally repeatable imaging method of retinal structures with micrometer resolution has considerably contributed to effective and comprehensive monitoring of the response to anti-VEGF treatment and managing treatment interval^19^.

However, determining a compound’s adverse effects still mostly relies on the histopathologic readout^20^. An important limitation is that such a histopathological examination can only be conducted once, so the longitudinal processes cannot be observed. Furthermore—and for obvious reasons—histology sampling cannot be performed on living humans without invasive and destructive procedures.

Although OCT does not allow for the assessment of all morphological parameters, such as the involved cell types or structural changes at the cellular/subcellular level, this technique has advanced the understanding of specific changes at the vitreoretinal interface and fluid changes in the retina. Furthermore, OCT enables the measurement of thickness variations in vivo and the longitudinal monitoring of findings^21,22^. Therefore, OCT imaging in preclinical studies is of unique translational importance to facilitate safety monitoring in clinical trials.

To date, there have been two major weaknesses in accompanying OCT imaging: on the one hand, only OCT data of a few cynomolgus monkeys are available^23,24^, and these data were mostly analyzed manually^25^. To overcome these limitations and further automate in vivo imaging readout capabilities—thus increasing both the sensitivity and objectivity of preclinical ocular safety assessments—in the current study a machine learning algorithm was developed for retina segmentation in cynomolgus monkeys.

Although the term fovea is widely utilized in the clinical context, another goal of the present study was to propose more objective criteria for defining the deepest site of the fovea on OCT images. Thus, a grader-independent and, therefore, objective method to determine the deepest point on OCT images within the fovea (referred to as the nulla) is proposed.

A huge reference database of macular OCT images from healthy cynomolgus monkeys was made available, and measures in animals of different geographical origins were compared.

Evidence has been found that the entire macular thickness may not be a suitable biomarker in every case because there may be considerable variations in retinal thickness, even within the same species.

## Results

### Summary statistics and visualizations

**Figure 1:**
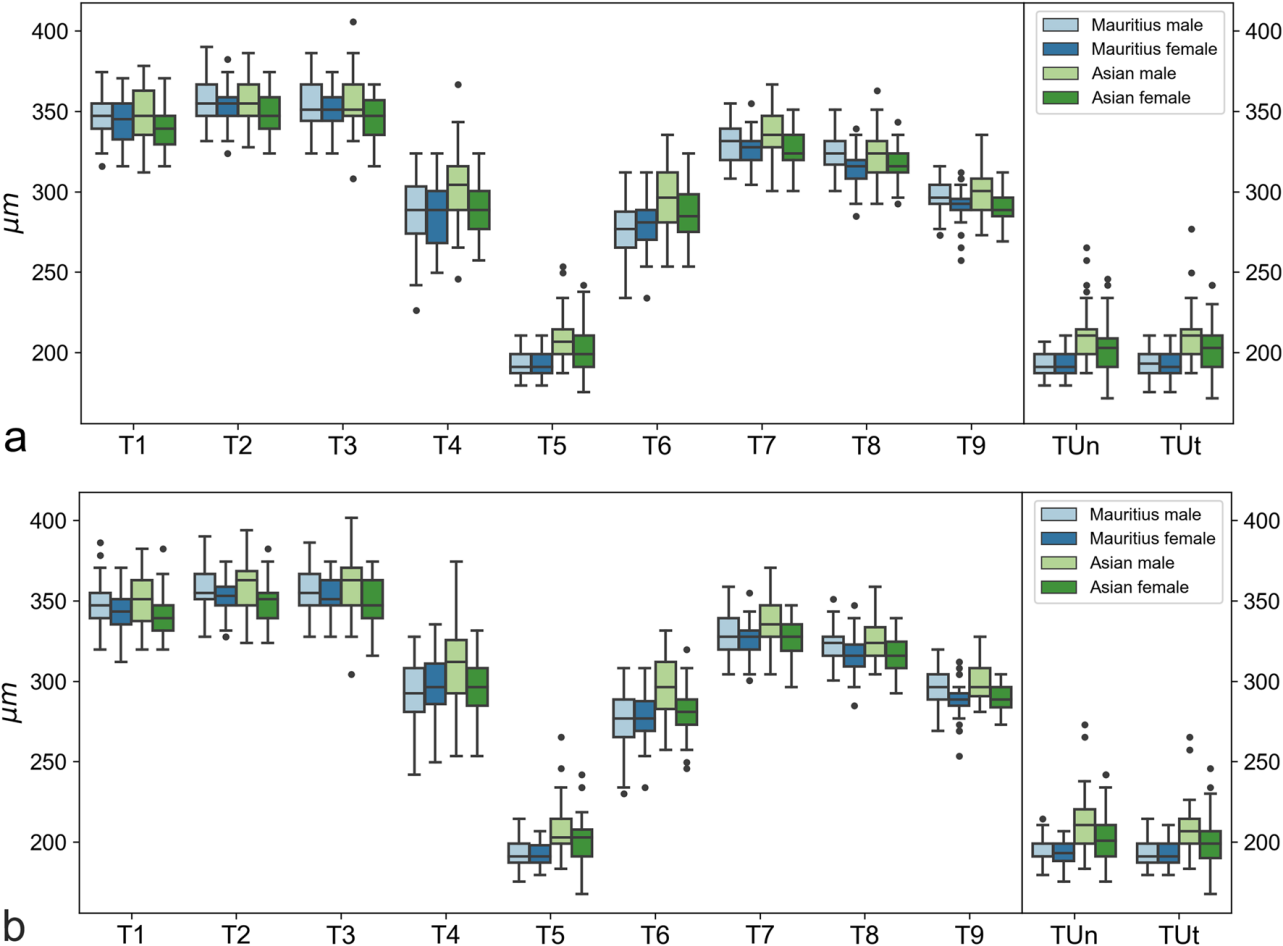
Boxplots showing the retina thickness values of the right (**a**) and left (**b**) eyes. For each thickness coefficient, numerical data of Mauritius male, Mauritius female, Asian male, and Asian female are plotted. Rectangular boxes represent interquartile ranges (IQR), which extend from Q1 to Q3. A black line in the middle of an IQR indicates a median. Upper whiskers extend to the last datum, which is smaller than Q3 + 1.5 × IQR. Lower whiskers extend to the first datum, which is greater than Q1 − 1.5 × IQR. Data beyond whiskers are outliers and plotted as black circles.

**Figure 2:**
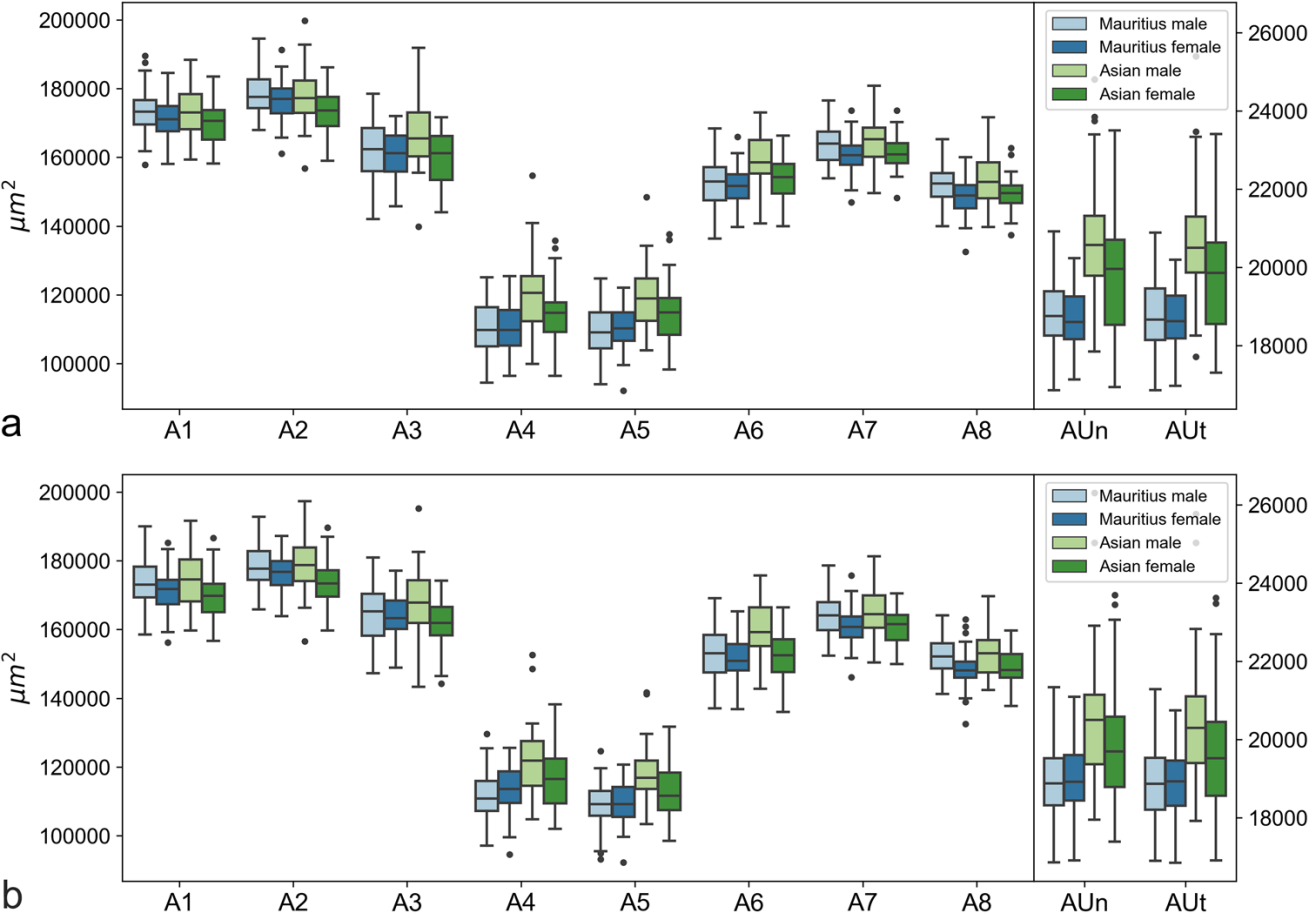
Boxplots of retina areas of the right (**a**) and left (**b**) eyes. For each area coefficient, numerical data of Mauritius male, Mauritius female, Asian male, and Asian female are plotted.

Retina thicknesses for the left and right eyes are shown as boxplots in Fig. 1. The boxplots roughly indicate the contour of the foveolar depression, with the smallest thicknesses at the nulla (T5). At the center (T4–T6), a difference between Asian and Mauritian macaques is apparent, with Asian macaques having larger retina thicknesses. Interestingly, at the edges (T1–T3, T7–T9), geographic origin does not seem to play a role, but females tend to have smaller retina thicknesses than males. The data at umbo strongly resemble data at T5. For the retina areas, the patterns are largely the same (Fig. 2). Tables 1, 2, and 3 present detailed summary statistics of all the coefficients. Summary statistics and boxplots are shown for the left and right eyes separately to avoid correlations between the eyes.

**Table 1:** Summary statistics of retina thickness values with respect to eye side, sex, and origin.

	Stats	Sex	Origin	T1	T2	T3	T4	TUn	T5	TUt	T6	T7	T8	T9
OD	Mean	Male	Mauritius	347.2	357.5	354.6	288.1	192.5	192.8	192.9	276.8	330.0	323.4	296.6
	Mean	Male	Asian	348.8	356.6	357.2	303.2	212.2	210.0	210.8	295.9	337.1	323.8	298.5
	Mean	Female	Mauritius	342.9	352.7	351.1	286.4	193.5	192.8	193.6	279.0	325.5	315.0	290.8
	Mean	Female	Asian	340.3	348.4	346.2	288.8	202.7	201.8	202.7	286.7	327.0	317.1	290.7
	Min	Male	Mauritius	315.9	331.5	323.7	226.2	179.4	179.4	175.5	234.0	308.1	300.3	273.0
	Min	Male	Asian	312.0	327.6	308.1	245.7	187.2	187.2	187.2	253.5	300.3	292.5	273.0
	Min	Female	Mauritius	315.9	323.7	323.7	249.6	179.4	179.4	175.5	234.0	304.2	284.7	257.4
	Min	Female	Asian	315.9	323.7	315.9	257.4	171.6	175.5	171.6	253.5	300.3	292.5	269.1
	Max	Male	Mauritius	374.4	390.0	386.1	323.7	206.7	210.6	210.6	312.0	354.9	351.0	315.9
	Max	Male	Asian	378.3	386.1	405.6	366.6	265.2	253.5	276.9	335.4	366.6	362.7	335.4
	Max	Female	Mauritius	370.5	382.2	374.4	323.7	210.6	210.6	210.6	312.0	354.9	339.3	312.0
	Max	Female	Asian	370.5	374.4	366.6	323.7	245.7	241.8	241.8	323.7	351.0	343.2	312.0
	Std	Male	Mauritius	13.2	13.0	13.5	20.5	7.6	7.1	8.4	16.9	11.7	10.5	9.8
	Std	Male	Asian	16.4	14.5	16.7	22.5	16.4	15.2	15.8	18.8	13.9	15.1	14.8
	Std	Female	Mauritius	14.1	12.2	10.5	19.5	8.0	7.7	8.0	15.4	10.8	11.5	11.3
	Std	Female	Asian	13.5	12.5	13.8	18.4	16.1	15.2	16.0	17.3	12.2	10.0	9.7
OS	Mean	Male	Mauritius	347.8	358.4	356.5	292.8	193.7	192.8	193.9	276.6	329.8	323.5	295.4
	Mean	Male	Asian	350.4	359.1	359.0	307.6	212.1	207.7	208.1	296.1	338.7	325.7	299.7
	Mean	Female	Mauritius	342.6	352.1	352.6	296.9	194.3	192.7	193.2	277.3	325.4	315.7	288.3
	Mean	Female	Asian	340.4	348.7	348.6	294.8	203.2	200.6	200.2	282.0	326.5	315.5	288.8
	Min	Male	Mauritius	319.8	327.6	327.6	241.8	179.4	175.5	179.4	230.1	304.2	300.3	269.1
	Min	Male	Asian	319.8	323.7	304.2	253.5	183.3	183.3	183.3	257.4	304.2	304.2	280.8
	Min	Female	Mauritius	312.0	327.6	327.6	249.6	175.5	179.4	179.4	234.0	300.3	284.7	253.5
	Min	Female	Asian	319.8	323.7	315.9	253.5	175.5	167.7	167.7	245.7	296.4	292.5	273.0
	Max	Male	Mauritius	386.1	390.0	386.1	327.6	214.5	214.5	214.5	308.1	358.8	351.0	319.8
	Max	Male	Asian	382.2	393.9	401.7	374.4	273.0	265.2	265.2	331.5	370.5	358.8	327.6
	Max	Female	Mauritius	370.5	374.4	374.4	335.4	206.7	206.7	210.6	308.1	354.9	347.1	312.0
	Max	Female	Asian	382.2	382.2	374.4	331.5	241.8	241.8	245.7	319.8	347.1	339.3	304.2
	Std	Male	Mauritius	13.0	12.8	13.3	19.1	7.3	7.2	8.3	16.5	12.8	10.4	10.5
	Std	Male	Asian	15.7	16.3	17.8	24.1	18.2	15.6	16.0	18.8	14.9	14.0	12.8
	Std	Female	Mauritius	14.3	11.1	11.6	19.0	7.4	7.1	8.2	16.0	11.6	12.1	11.5
	Std	Female	Asian	13.6	12.6	14.3	17.9	15.1	15.7	15.9	16.0	11.7	10.1	8.4

Values are presented in µm.

*OD* oculus dexter, *OS* oculus sinister, *Std* statistical analysis, *U* umbo, *n* nasal, *t* temporal.

**Table 2:** Summary statistics of retina areas with respect to eye side, sex, and origin.

	Stats	Sex	Origin	A1	A2	A3	A4	AUn	AUt	A5	A6	A7	A8
OD	Mean	Male	Mauritius	173,388	178,351	162,236	110,407	18,836	18,786	109,552	152,737	164,084	152,379
	Mean	Male	Asian	173,998	178,024	166,776	120,136	20,732	20,675	119,222	159,323	164,887	153,467
	Mean	Female	Mauritius	170,944	176,225	161,037	110,488	18,746	18,717	110,469	151,820	160,663	148,861
	Mean	Female	Asian	169,848	173,579	159,915	114,358	19,881	19,874	115,285	153,931	161,011	149,622
	Min	Male	Mauritius	157,796	167,913	142,089	94,408	16,858	16,858	94,026	136,334	153,925	140,003
	Min	Male	Asian	159,409	156,826	139,863	99,916	17,847	17,714	103,829	140,755	149,613	139,719
	Min	Female	Mauritius	158,091	161,072	145,803	96,404	17,133	16,961	92,123	139,726	146,988	132,579
	Min	Female	Asian	158,158	158,995	144,027	96,402	16,933	17,307	98,310	140,008	148,236	137,386
	Max	Male	Mauritius	189,582	194,555	178,505	125,060	20,919	20,889	124,778	168,413	176,493	165,275
	Max	Male	Asian	188,342	199,738	191,925	154,682	24,808	25,394	148,440	173,061	180,890	171,661
	Max	Female	Mauritius	184,587	191,286	172,045	125,496	20,229	20,192	122,035	165,992	173,616	160,087
	Max	Female	Asian	183,525	186,157	171,658	135,746	23,504	23,408	137,657	166,361	173,639	162,680
	Std	Male	Mauritius	6517	6584	7937	7071	936	921	6518	6588	5497	4855
	Std	Male	Asian	7473	7897	8862	10,254	1588	1506	9099	7199	6924	7579
	Std	Female	Mauritius	6353	5773	6326	7323	788	827	6349	5534	5442	5830
	Std	Female	Asian	6617	6278	7734	8582	1652	1633	8644	6877	4967	4952
OS	Mean	Male	Mauritius	173,852	178,694	164,033	111,689	18,935	18,881	109,246	152,827	164,143	152,132
	Mean	Male	Asian	174,620	179,050	168,332	121,453	20,640	20,494	117,960	160,010	165,783	153,456
	Mean	Female	Mauritius	170,989	176,601	163,530	113,695	19,036	18,928	109,380	151,333	160,857	148,438
	Mean	Female	Asian	169,996	173,783	161,583	116,636	19,862	19,705	112,829	153,015	160,675	148,970
	Min	Male	Mauritius	158,594	165,849	147,303	97,092	16,858	16,895	93,134	137,082	152,414	141,270
	Min	Male	Asian	159,752	156,594	143,334	104,732	17,950	17,911	103,378	142,745	150,388	142,451
	Min	Female	Mauritius	156,251	163,843	148,892	94,568	16,902	16,844	92,213	136,807	146,109	132,522
	Min	Female	Asian	156,710	159,658	144,272	101,938	17,387	16,906	98,550	136,025	149,916	137,714
	Max	Male	Mauritius	190,039	192,769	180,932	129,639	21,332	21,284	124,607	169,060	178,684	164,069
	Max	Male	Asian	191,610	197,312	195,304	152,599	26,303	25,763	141,757	175,729	181,264	169,739
	Max	Female	Mauritius	185,208	187,214	177,139	125,504	21,092	20,741	120,651	165,333	175,781	163,017
	Max	Female	Asian	186,704	189,637	174,242	138,178	23,695	23,620	131,750	166,401	170,465	159,715
	Std	Male	Mauritius	6456	6247	7487	6535	948	990	6142	6747	5795	5158
	Std	Male	Asian	8084	8277	10,029	10,467	1663	1621	8580	8166	6870	6755
	Std	Female	Mauritius	6414	5177	7107	6764	923	898	6241	6466	5481	6091
	Std	Female	Asian	6759	6404	7669	8105	1537	1581	8072	6688	5217	4482

Values are presented in µm^2^.

*OD* oculus dexter, *OS* oculus sinister, *Std* statistical analysis, *U* umbo, *n* nasal, *t* temporal.

**Table 3:** Summary subfield analysis of umbo retina thickness and area values.

	OD			OS		
	TUt	Nulla	TUn	TUt	Nulla	TUn
**A. Males**						
Mean	202	201	202	201	200	202
Min	176	179	179	179	176	179
Max	277	254	265	265	265	273
Std	16	15	16	15	14	16

	AUt		AUn	AUt		AUn
Mean	19,700		19,753	19,642		19,718
Min	16,858		16,858	16,895		16,858
Max	25,394		24,808	25,763		26,303
Std	1611		1653	1599		1608

	OD			OS		
	TUt	Nulla	TUn	TUt	Nulla	TUn
**B. Females**						
Mean	199	198	198	197	197	199
Min	172	176	172	168	168	176
Max	242	242	246	246	242	242
Std	14	13	14	13	13	13

	AUt		AUn	AUt		AUn
Mean	19,335		19,352	19,306		19,438
Min	16,961		16,933	16,844		16,902
Max	23,408		23,504	23,620		23,695
Std	1451		1448	1344		1335

	OD			OS		
	TUt	Nulla	TUn	TUt	Nulla	TUn
**C. Mauritius**						
Mean	193	193	193	194	193	194
Min	176	179	179	179	176	176
Max	211	211	211	215	215	215
Std	8	7	8	8	7	7

	AUt		AUn	AUt		AUn
Mean	18,762		18,804	18,899		18,972
Min	16,858		16,858	16,844		16,858
Max	20,889		20,919	21,284		21,332
Std	894		892	962		945

	OD			OS		
	TUt	Nulla	TUn	TUt	Nulla	TUn
**D. Asian**						
Mean	207	206	208	204	204	208
Min	172	176	172	168	168	176
Max	277	254	265	265	265	273
Std	17	16	17	17	16	17

	AUt		AUn	AUt		AUn
Mean	20,303		20,337	20,115		20,267
Min	17,307		16,933	16,906		17,387
Max	25,394		24,808	25,763		26,303
Std	1626		1683	1660		1661

Values for thickness are in µm; values for surface area in µm^2^.

*A* area, *T* thickness, *U* umbo, *n* nasal, *t* temporal.

### Statistical analyses

#### Correlation analysis

Columns 1 to 8 in Table 4 reveal that the retina thickness and corresponding area coefficients (e.g., T1 and A1) are highly correlated (correlations between 0.89 and 0.96). Moreover, columns 9 to 12 in Table 4 show that all four umbo coefficients are highly correlated with T5 (correlations between 0.92 and 0.96). Regarding the correlations among the nine thickness coefficients of T1–T9, Table 5 demonstrates the generally rather high correlation between adjacent coefficients (0.55–0.91). However, for nonadjacent coefficients, this correlation is smaller (0.07–0.85). For statistical analyses, we considered only the nine thickness coefficients T1–T9, excluding the eighth area (A1–A8) and four umbo (TUn, TUt, AUn, and AUt) coefficients.

**Table 4:** Pearson correlation among thickness and area coefficients.

	1	2	3	4	5	6	7	8	9	10	11	12
Var1	T1	T2	T3	T4	T6	T7	T8	T9	T5	T5	T5	T5
Var2	A1	A2	A3	A4	A5	A6	A7	A8	TUn	TUt	AUn	AUt
Corr	0.96	0.94	0.91	0.89	0.90	0.90	0.91	0.95	0.96	0.95	0.92	0.92

High correlations exist between thickness and corresponding area coefficients (e.g., T1 and A1) and between the four umbo coefficients (TUn, TUt, AUn, and AUT) and thickness T5.

**Table 5:** Pearson correlation matrix for thickness coefficients T1–T9.

	T1	T2	T3	T4	T5	T6	T7	T8	T9
T1	1	0.91	0.71	0.35	0.07	0.29	0.55	0.63	0.58
T2		1	0.84	0.43	0.07	0.35	0.66	0.68	0.60
T3			1	0.71	0.25	0.59	0.79	0.58	0.46
T4				1	0.54	0.83	0.64	0.35	0.29
T5					1	0.59	0.33	0.10	0.13
T6						1	0.70	0.31	0.27
T7							1	0.74	0.61
T8								1	0.90
T9									1

Adjacent coefficients (e.g., T1 and T2) generally show a higher correlation than nonadjacent coefficients (e.g., T1 and T3). The lower triangle of the matrix has been left empty because the correlation matrix is symmetric.

#### Principal component analysis

**Figure 3:**
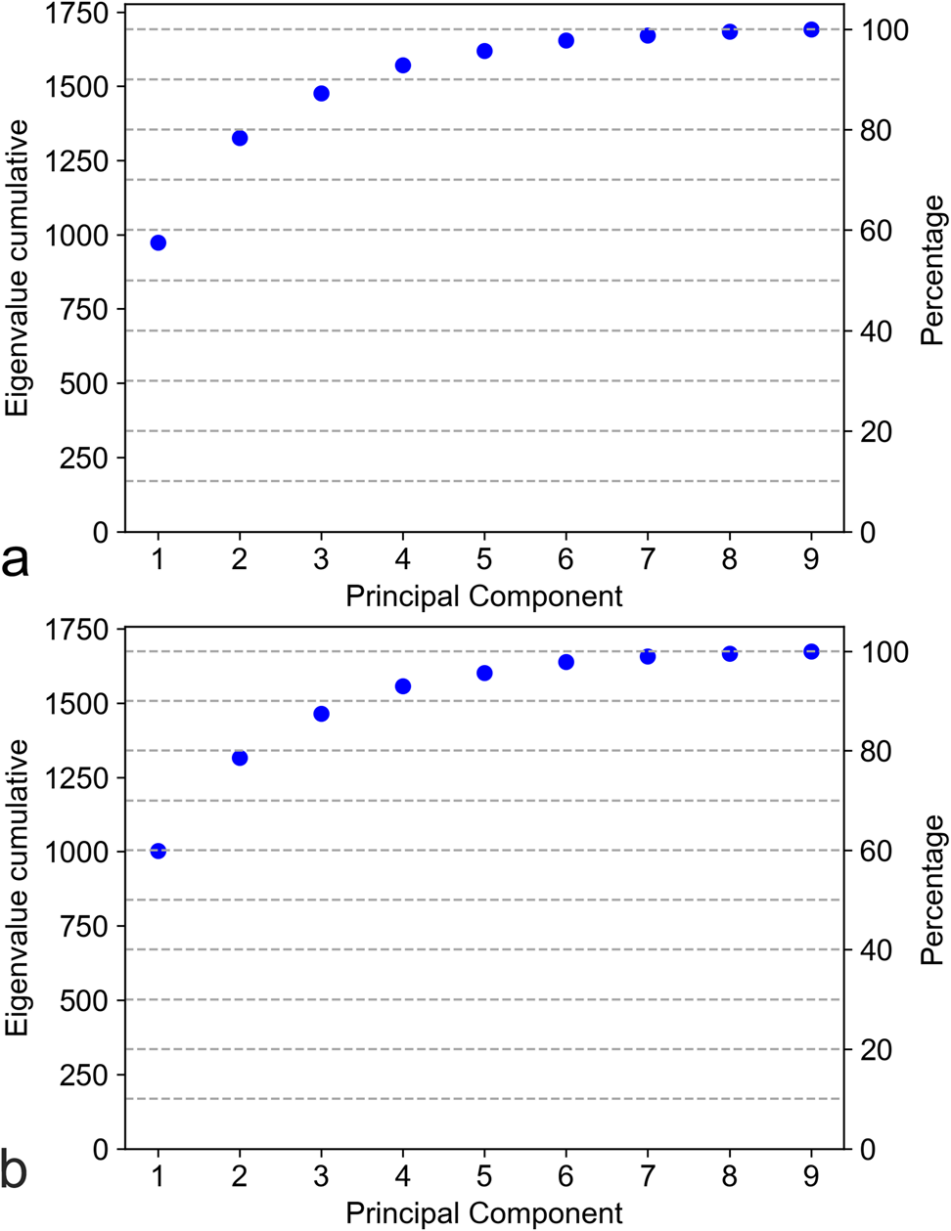
Principal component analysis (PCA) scree plots showing the cumulative eigenvalues of the nine principal components (PCs) for the right (**a**) and left (**b**) eyes. Eigenvalues correspond to the explained variability of the respective PC. The first three PCs explain 87.5% and 87.3% of the variability for the left and right eyes, respectively.

A principal component analysis (PCA) was used to investigate the latent factors that could explain the variability in the thickness parameters T1–T9. The plots in Fig. 3 reveal that the first three principal components (PCs) explain 87.5% and 87.3% of the variability for the left and right eyes, respectively. That is, the first three PCs mainly determine the values of T1–T9. Table 6 shows the PCA coefficients of the first three PCs for the left and right eyes. The patterns for the left and right eyes are largely the same. The first PC is a weighted average of the nine thickness parameters, with the weights roughly corresponding to the relative size of each thickness. That is, the first PC is an overall thickness factor. The second PC appears to be considering the thickness values at the edges (negative sign) versus those at the center (positive sign). This suggests that the second PC is a center-vs.-edge factor. The third PC seems to be considering nasal versus temporal thickness parameters (mainly reversed signs for PCA coefficients left and right of T5). That is, the third PC is a nasal-vs.-temporal factor.

**Table 6:** Principal component coefficients of the first three principal components for left and right eyes.

PC	Eye	T1	T2	T3	T4	T5	T6	T7	T8	T9
1	Left	10.5	11.6	12.2	10.3	5.4	10.0	12.3	11.2	9.8
2	Left	− 5.6	− 4.8	0.2	7.2	9.7	7.9	1.1	− 4.9	− 5.2
3	Left	− 3.9	− 4.0	− 4.5	− 1.5	3.7	− 0.4	1.1	5.2	7.2
1	Right	10.8	11.6	12.3	9.9	5.1	9.0	12.2	10.7	10.1
2	Right	− 5.2	− 4.8	0.5	7.9	9.7	9.0	1.2	− 5.8	− 5.5
3	Right	4.0	4.4	4.7	1.3	− 3.8	− 0.1	− 1.6	− 5.6	− 6.6

Principal component analysis (PCA) coefficients of the first three principal components (PCs). The table shows the PCA coefficients for the left (three top rows) and right (three bottom rows) eyes. The patterns are largely the same for the left and right eyes. The first PC is an overall thickness factor with PCA coefficients of T1–T9 corresponding roughly to the relative size of the respective thickness. The second PC is a center-vs.-edge factor. The third PC is a nasal-vs.-temporal factor. The sign of the third PC is mostly reversed for the left and right eyes.

The multivariate analysis of variance (MANOVA) (Table 7) demonstrates that the independent variables of sex and origin have a significant impact on the dependent variables T1–T9 in both eyes. The effect of origin was stronger than the effect of sex. The interaction terms between sex and origin were not significant and, thus, were removed from the final models.

**Table 7:** Multivariate analysis of variance (MANOVA) results.

Eye	Variable	Wilks’ lambda	Pr > F
Left	Sex	0.8142	5.9e−5
Left	Origin	0.6454	1.9e−12
Right	Sex	0.8650	3.1e−3
Right	Origin	0.6257	1.4e−13

A MANOVA was performed for the left (first two rows) and right (bottom two rows) eyes separately. The independent variables of sex and origin have an effect on the dependent variables T1–T9 in left and right eyes. The effect of origin is stronger than the effect of sex. Effect size is measured using Wilks’ lambda. The test results are equivalent with Pillai's trace, Hotelling-Lawley trace, and Roy's greatest root.

#### Analysis of variance

Table 8 shows the results of the nine two-way analysis of variance (ANOVA) analyses. Differences in sex are significant for the outer thickness parameters (T1–T3 and T7–T9), whereas differences in origin are significant for inner thickness values (T5 and T6). Interaction terms between sex and origin are not significant and, thus, have been removed from the final models.

**Table 8:** Summary of *p*-values in two-way analysis of variance (ANOVA) for measured retinal thickness parameters in left and right eyes in relation to sex and origin.

Thickness	T1	T2	T3	T4	T5	T6	T7	T8	T9
Sex	9.8e−04**	1.3e−04**	2.8e−03*				1.4e−04**	1.7e−06***	5.2e−07***
Origin					5.1e−10***	1.8e−06***	6.4e−03		

Thickness	T1	T2	T3	T4	T5	T6	T7	T8	T9
Sex	6.1e−03	2.6e−03*	1.9e−0*				3.1e−04**	8.9e−05***	3.0e−04**
Origin				3.6e−03	1.4e−12***	3.6e−07***			

Three stars indicate *p*-values < 0.001/9. Two stars indicate *p*-values < 0.01/9. One star and one dot indicate *p*-values < 0.05/9 and < 0.1/9, respectively. Nine is the number of hypotheses and, thus, the factor applied to adjust significance levels (Bonferroni correction). Exact *p*-values are only shown if the results are significant.

## Discussion

The main aim of the current study was to generate reference data on a large number of cynomolgus macaque eyes (*M. fascicularis*)^26,27^, which, because of their genetic similarity to humans, have been successfully introduced into biomedical research^28–33^. Alongside histopathologic examinations^31,34^, OCT has been demonstrated as a useful imaging tool to assess ocular toxicity, such as for ocular inflammation, in preclinical studies^24,33,35,36^.

Despite all the successes, OCT remains a fairly new technique in animal research, which may account for the lack of reference data. In addition, the analysis of OCT scans from cynomolgus macaques is time-consuming and associated with undesired deviations because of the often manually—thus relatively arbitrary—performed readings of the values^37,38^. Consequently, interpreting such OCT results in the context of the natural variability of macular thickness is impossible.

To overcome these limitations and offer more in-depth knowledge compared with our previous report^39^, the present study successfully implemented an automatic machine learning application that was supplemented with a classic approach in a hitherto unprecedented number of cynomolgus monkeys. Their retinal thickness at the fovea—or the site of highest visual acuity—was assessed to provide reference values. Despite morphological similarities among the eyes of most primates, the obtained data show that in addition to the known differences in the cone numbers^40–42^, corresponding variations can also be found at the structural OCT level. These variations occur even within the same species of different geographic origins, which are commonly used in preclinical toxicology studies^43^. This circumstance was also evident in the subanalysis of the umbo area, that is, the site with the highest density of photoreceptors (cones). The integrity of this zone is essential for the best visual acuity, and its disintegration can be the first sign of various diseases, such as age-related macular degeneration, but also, for example, the formation of a macular hole^21^. Our data suggest that despite being the same species, certain morphological characteristics have evolved differently. It is important to be aware of this fact, particularly if, during drug development, researchers decide to alternate trials between monkeys of different origins. Consequently, both origins cannot necessarily be used interchangeably in each use case. This particular difference in macular morphology would be the most relevant if we relied on quantitative macula measurements.

The metabolic differences between Asian and Mauritian macaques have been investigated and described previously^42^. As an example, it has been shown that in Mauritius macaques, the retroviral restriction factor TRIMCyp is not present, which is in contrast to a higher expression of TRIMCyp in Indonesian macaques, causing methodological challenges for AIDS research^44^. However, so far, no data have been available for ocular structure.

In agreement with a previous study, there was no significant difference in the central depression (T4, T5, and T6) between the sexes^39^. Interestingly, individual ANOVA analyses of the data show significant differences in nasal and temporal thickness values (T1–T3 and T7–T9, respectively) between male and female individuals. This suggests that the averaged values of macula measurements, which are commonly used in ophthalmic research and obtained over a larger range, underestimate local variations, so caution is advised in interpreting these values. Overall, females showed a slightly thinner retina than males—which is similar to humans—although major differences between the provenances and age might also have to be taken into account^45–47^.

The mean overall retina thickness in the present study was 199 µm, compared with 244 µm in cynomolgus macaques^39^ and 305 µm in humans^48^. This deviation can be explained by the fact that in comparable cynomolgus monkey studies, the value was determined over a larger subfield, which is in contrast to the current subfield study that only calculated the values from the deepest point within the foveola. Regarding minimal foveal thickness, an unpublished study (Vilupuru A. Optical coherence tomography in normal eyes of rhesus monkeys. American Academy of Optometry, 2005) using Stratus time-domain OCT (TD-OCT) measured a value that was approximately 60 µm lower. However, considerable deviations between generations of OCT devices and better reproducibility for the spectral-domain system used in the present study have been reported^49,50^. Furthermore, the number of animals was several times higher in the present study. In summary, a final appreciation of the mentioned TD-OCT data is not feasible because the methodology and values have not been made publicly available in full detail.

The current study may be limited because the axial length and refractive status were not known, and diurnal variations have not been measured yet. However, such limits are often inherent in retrospective studies and can be complemented in future prospective studies. Of course, the definition of the reference position nulla can be a point of discussion and may represent an additional limit to naturally occurring variations^51^. Using the suggested fully automatic image processing method, human selection bias with regard to the position of nulla can be largely eliminated or even prevented. Another limitation could be that in the accumulated database, no repeated retina measurements were available. However, for repeated retina OCT measurements, only minor variations were found that were within the resolution and repeatability range of the OCT devices^52,53^.

The current number of B-scans may not have captured the effective fovea center in every single eye. In addition, the analyzed region does not correspond to the whole retina, so conclusions can only be derived for the central retina. Nevertheless, the reference database covers the area of greatest cell density^54,55^.

In summary, for the first time, the current study succeeded in applying a hybrid machine learning approach to a large number of macaque eyes to determine the reference values for retinal thickness at the fovea. In addition, the present study has described the morphological variability of retinal thickness and how it relates to sex and the origin of these monkeys. Most importantly, the present study has shown that a thorough awareness of the constraining elements in model species supports the careful selection of the appropriate models for ophthalmic research and appropriate reading of the obtained data. The data provided are important for earlier and more sensitive detection, quantification, and characterization of toxic ocular effects in preclinical safety studies^56,57^. In particular, noninvasive OCT examinations can constitute an additional imaging method for comparative studies between OCT and histopathology^58,59^. However, compared with histology^60^, which is usually performed only once, OCT can be performed within seconds and as often as needed, all without damaging the ocular tissue or bloodstream. Consequently, OCT appears to be an ideal instrument for longitudinal investigations and can presumably enable better characterization and monitoring of lesions.

## Methods

### Animals

All experiments were performed in accordance with the relevant guidelines and regulations. Data were acquired from healthy, untreated cynomolgus monkeys (*Macaca fascicularis)* during ocular baseline examinations of routine pharmaceutical product development studies. Thus, no additional animals were used specifically for the current study. Animal care and experimentation were in accordance with the guidelines of the Association for Assessment and Accreditation of Laboratory Animal Care (AAALAC), ARRIVE guidelines, the US National Research Council, and/or the Canadian Council on Animal Care (CCAC). The studies were approved by one of the following Institutional Animal Care and Use Committees (IACUCs): Charles River Laboratories Montreal, ULC Institutional Animal Care and Use Committee (CR-MTL IACUC), IACUC Charles River Laboratories Reno (OLAW Assurance No. D16-00594), and Institutional Animal Care and Use Committee (Covance Laboratories Inc., Madison, WI) (OLAW Assurance #D16-00137 (A3218-01)). The protocols for the original drug development studies were reviewed and approved by the respective Institutional Animal Care and Use Committees of their respective contract research organizations.

The animals were group-housed in stainless steel cages according to European housing standards (Annex III of Directive 2010/63/EU). The temperature of the animal room was maintained between 20 and 26 °C, with humidity between 30 and 70%. The light cycle was 12 h light and 12 h dark, except during designated procedures. The animals were fed a standard diet of pellets supplemented with fresh fruits and vegetables. Water treated with reverse osmosis and ultraviolet irradiation was freely available to each animal via an automated watering system. Psychological and environmental enrichment was provided to the animals, except during study procedures and activities. The animals were purpose-bred and of Mauritian or mixed Asian origin (the exact geographical location for the latter is unknown).

OCT scans were obtained from 374 eyes of 203 animals. Information regarding age was available for 159 subjects, with an overall mean age of 4.98 years (range 2.5–5 years). Females contributed 147 eyes (39.30%) and males 227 eyes (60.70%); of the total eyes, 186 were left eyes (47.74%), and 188 (50.26%) were right eyes. Regarding the geographic region, 199 eyes originated from Mauritius (53.20%), and 159 eyes were derived from Asia (46.80%). Sixteen eyes were of unknown origin, but the sex was defined.

### Inclusion and exclusion criteria

The inclusion criteria were healthy and untreated cynomolgus monkeys derived from Mauritius or Asian origin who were between 30 and 50 months of age and weighed between 2.5 and 5.5 kg. Only healthy eyes, with optically clear ocular media and no observed anterior or posterior segment pathologies, were included in the study. Eyes of an undocumented sex, origin, or eye side were excluded from the subanalyses.

### OCT imaging

OCT was performed under general anesthesia with dilated pupils using spectral-domain OCT on the Spectralis HRA + OCT Heidelberg platform (Heidelberg Engineering, Heidelberg, Germany) as reported^37,39^. The same scan protocol was selected for all animals with a horizontal line scan pattern (centered over the fovea) with a size of 20° × 20°, 25 raster lines separated by 221 μm (scan length 5.3 mm, 512 × 496 pixels, scan depth 1.9 mm). The data were exported directly from the OCT device as original B-scan files in a bitmap image data (BMP) format using the manufacturer’s software.

### The fovea position–location problem

Because the examined animals for obvious reasons could not follow the instructions of the operator to exactly and steadily fixate on a presented target, the determination of the fovea center by an OCT operator could be relatively arbitrary. The use of subjective criteria for the definition of the fovea may affect the preciseness of a measurement^61^. Although the term fovea is frequently used in the clinical context, it does not represent a precise anatomical term or position, so the determination of fovea landmarks may be associated with uncertainties^55,62^.

**Figure 4:**
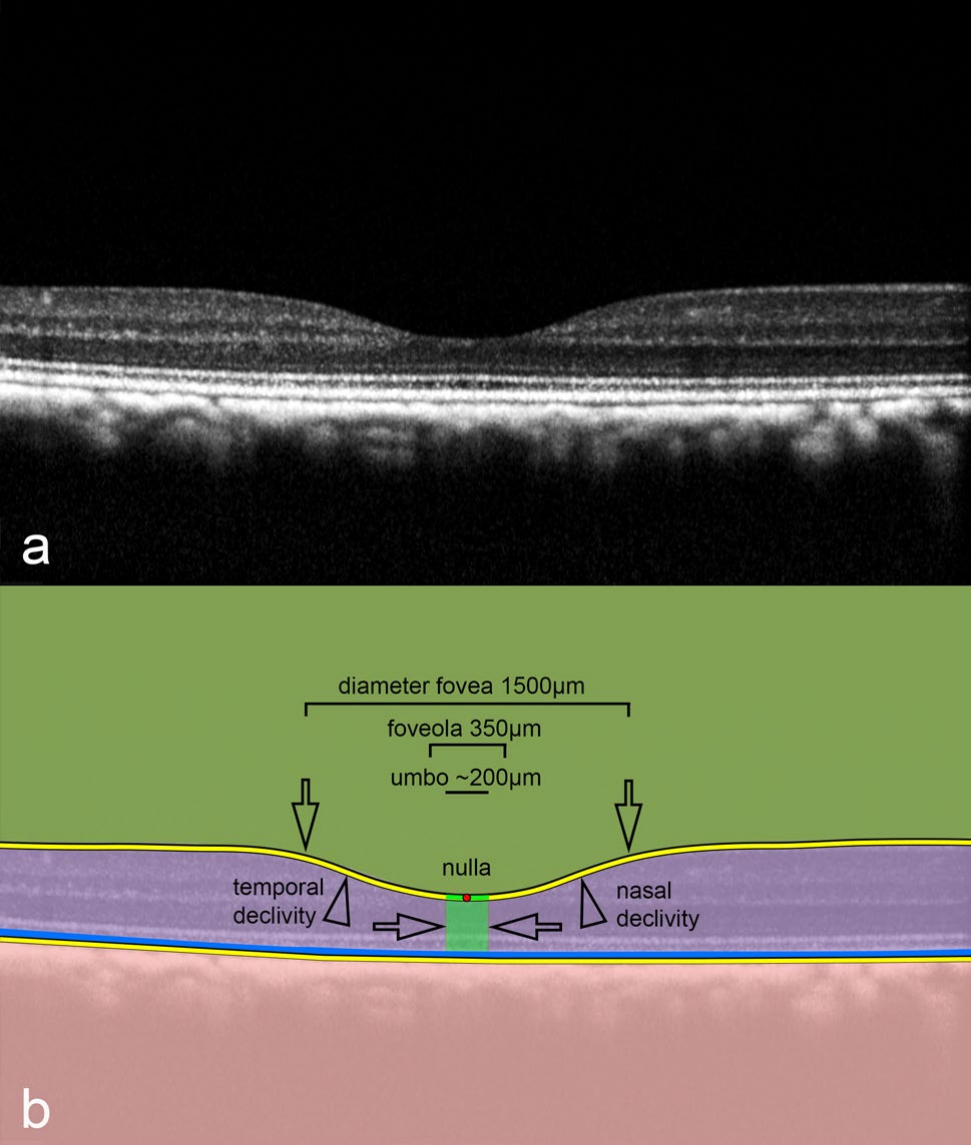
Definition of anatomical foveolar landmarks. (**a**) A cross-sectional optical coherence tomography (OCT) of the right concaviclivate fovea in a healthy macaque is shown. (**b**) A schematic overlay of the same scan illustrating the anatomical landmarks. The area of the fovea is characterized as a central depression (single arrows) descending in a more or less symmetrical curvilinear shape (double arrowheads) to the bottom, which is named the foveola. The deepest location inside the fovea is determined as the nulla (marked as a red dot) and represents an OCT-based anatomical landmark. The umbo in the OCT image is designated as the proposed center of the fovea (highlighted as the green area between the two arrows) near the nulla at a distance of 100 µm to each side. Based on these conventions, the following OCT-based sequence can be proposed: macula lutea > perifovea > parafovea > fovea > foveola > umbo > nulla. The retinal pigment epithelium is highlighted in blue.

Currently, a concaviclivate fovea (fovea from the Latin “ditch” or “pit”) represents a depression of the center of the retina^9^ (Fig. 4). Thereby, a supposed margin can be assumed, which transits into a descending clivus or slope (clivus from the Latin “slope” or “hill”), resulting in a certain bowl-shaped configuration. The base is referred to as the foveola or “little fovea.” Although the umbo is interpreted clinically as the center of the fovea, such a definition proves to be rather problematic in cross-sectional imaging in animals because the geometry and extension of the fovea differ remarkably among different species^55^.

### Definition of nulla as an OCT structure-based fovea parameter

The fovea can be visualized very well with an excavation of the central retina on OCT imaging (Fig. 4a). To eliminate the position–location problem of the fovea toward an objective assessment and in a slight customization to previous reports^51,55^ where the deepest point of the foveal pit was indicated, a modified interpretation of the deepest position within the retina is proposed based on structural OCT information.

For this purpose, an automatic fovea depression contour finder approach was pursued, in which the definition of the particular fovea center (displayed on the OCT-B scan) was defined as the deepest point within the foveolar cavity (Fig. 4b). The resulting position was referred to as the nulla, which implies that on the particular B-scan or a stack of B-scans no deeper excavation could be present below the nulla within a fovea. The retinal thickness at the nulla position corresponds to the thinnest retinal thickness within the pit or inner fovea, where the incident light can interact most directly with the photoreceptors. This position holds an important value because the area within 150–200 µm around the nulla represents a crucial site containing the highest concentration of photoreceptors (cones)^55^.

### Semantic image segmentation

**Figure 5:**
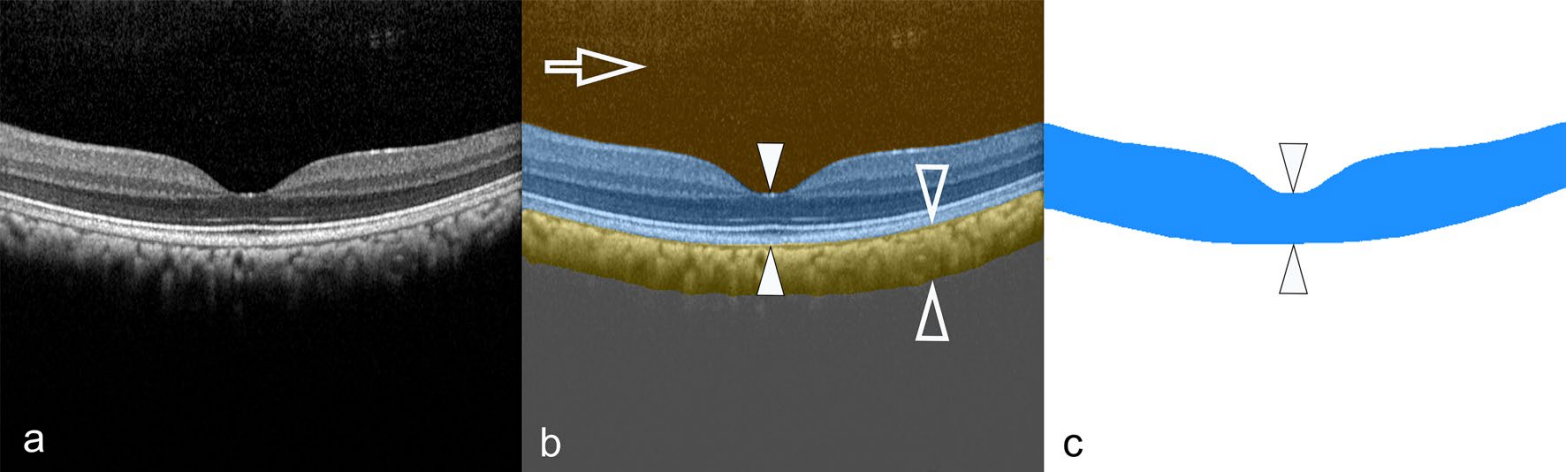
Illustration of the semantic compartment annotations. (**a**) An original cross-sectional B-scan was exported for (**b**) automatic machine learning compartmentalization of the posterior eye segment into the vitreous (arrow, marked in brown), retina (double-closed arrowhead, marked in blue), and choroid (double-open arrowhead, marked in orange). (**c**) The resulting retina component (blue, double-open arrowhead) was used for further analyses in one and two dimensions.

**Figure 6:**
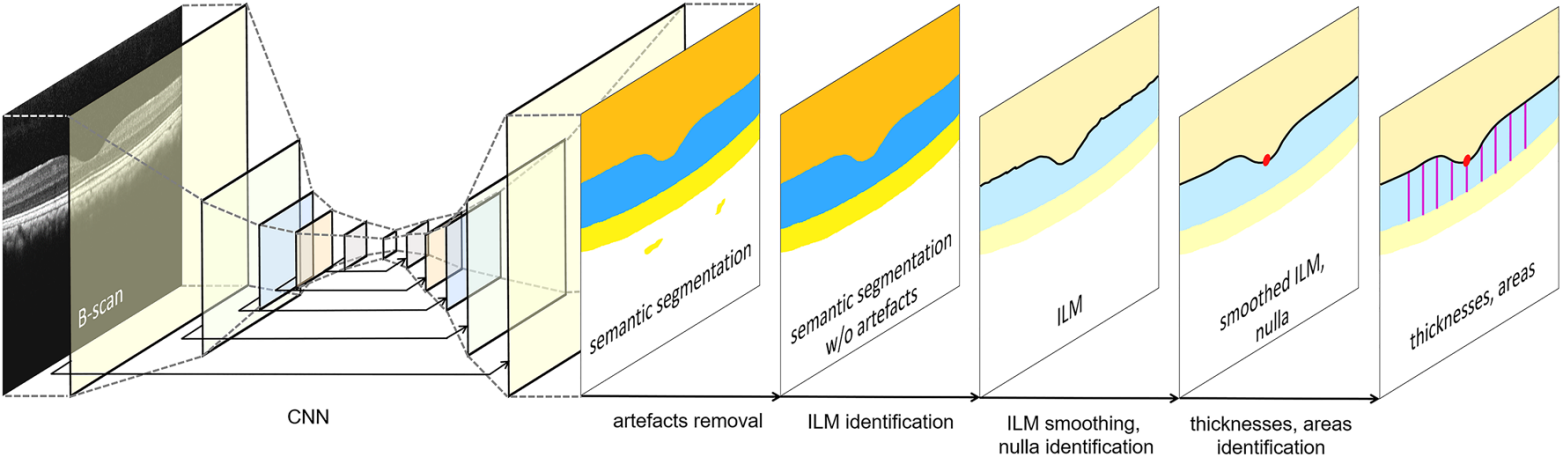
Illustration of the image processing pipeline that consists of six steps. First, a raw B-scan is used as input to a modified U-Net^64^. The output is a semantic segmentation map. In between, 11 network layers are schematically visualized, including the network’s five skip connections (indicated by black arrows)^61,63^. Second, segmentation artifacts are removed. Third, the region of the internal limiting membrane (ILM) separating vitreous from retina is identified (black line). Fourth, the ILM is smoothed (black line). Fifth, the nulla (red dot) is identified on the smoothed ILM. Sixth, retina thicknesses and areas are measured (violet bars).

The algorithm developed for the objective identification of the nulla and subsequent measurement of retina thicknesses and areas consists of six steps, which are illustrated in Figs. 5 and 6. The first step is the generation of pixel-wise semantic segmentation maps of the retina compartment using a convolutional neural network (CNN) for each B-scan (Fig. 5). The CNN was developed and described in detail in a previous study^63^. In summary, it uses a modified U-Net architecture^64^ with 22 convolutions, 5 transposed convolutions, and 5 skip connections, which have previously been shown to also be highly effective in learning semantic segmentation maps from human OCT B-scans^61^. The CNN was trained and validated on a representative subset of the OCT cynomolgus monkey data set^39^ used in the current work. This subset—the ground truth—consisted of a total of 1100 B-scans (44 eyes from 44 individuals, each eye contributing 25 B-scans). For each individual, either the left or right eye was included. The ground truth was annotated by three experienced and independent retina specialists and randomly split into training, validation, and test sets of 675, 225, and 200 B-scans, respectively. For the training and validation sets, each human grader annotated 225 and 75 different B-scan, respectively. The test set of 200 B-scans was annotated by each human grader to investigate the intergrader agreement of the ground truth labels. Training set data were augmented by applying to each B-scan (1) vertical mirroring and (2) adding a random rotation between − 8° and 8° degrees, increasing the training set size to 2025 B-scans. Regarding CNN training, the model parameters were initialized^65^ and learned by minimizing an unweighted pixel-wise cross-entropy loss summed over the entire input. Adam optimization was used on a single NVIDIA TITAN-X GPU^66,67^. An initial learning rate of 6 × 10^−5^ and a mini-batch size of eight images was chosen, which had proven suitable in preliminary experiments. Training was stopped after 1920 iterations (7.6 epochs) after the validation set accuracy reached a plateau. On the test set, the differences between the CNN’s predictions and the annotations of the three human graders were, on average, smaller than the intergrader differences. The algorithm’s input are B-scans (Fig. 5a), here rescaled to 512 × 512 pixels and with its output corresponding pixel-wise semantic segmentation maps of the compartments vitreous, retina, choroid, and sclera (Fig. 5b). For the current study, only the retina compartment segmentations were further processed (Fig. 5c). The retinal inner boundary was defined as the transition from the hyporeflective vitreous to the hyperreflective retinal nerve fiber layer, more specifically in the area of the ILM. The retinal outer border was defined as the outer demarcation zone of the hyperreflective retinal pigment epithelium, just above the hyporeflective zone of the choriocapillaris. Detailed CNN architecture is shown in Fig. 2 of Maloca et al. (2019)^68^. See the previous study for further details about ground truth annotation, CNN training, and CNN evaluation^63^.

### Segmentation artifact removal

The second algorithmic step is the removal of segmentation artifacts. A small number of semantic segmentation maps contained small patches of misclassified regions, that is, segmentation artifacts. The CNN output in Fig. 6 shows an example of two such misclassified choroid regions in the sclera compartment. These segmentation artifacts were corrected by the following approach: in a segmentation map, the four main compartments (vitreous, retina, choroid, and sclera) are expected to form large, connected regions. This is true even in the presence of segmentation artifacts. Thus, the four largest connected regions generally correspond to the vitreous, retina, choroid, and sclera compartments. If additional connected regions are present, they must represent segmentation artifacts. If a segmentation artifact is completely surrounded by a compartment, the artifact can be removed by replacing it with the label of the surrounding compartment. Because the segmentation artifacts were small and did not occur at the compartment borders, they were effectively removed using this approach.

### Nulla identification

The third algorithmic step is the identification of the ILM (the border between vitreous and retina) from the semantic segmentation map (Fig. 6). This approach generally yielded noisy ILMs, which did not allow for a reliable identification of the lowest point: the nulla. Therefore, in a fourth algorithmic step, the identified ILM was smoothed using a moving average, with the moving average window being applied in two dimensions simultaneously: in the B-scan width dimension (window size 11) and B-scan “stack” dimension (window size 5). Afterwards, the fifth algorithmic step identified the lowest point on the smoothed internal limiting membrane  (Fig. 6). If multiple lowest points were found (usually adjacent), the coordinates of their center of mass were used as the lowest point and, if necessary, rounded to the coordinates of the voxel nearest to it. By utilizing this approach, the “central” B-scan was identified, along with the pixel coordinates of the nulla.

### Retinal thicknesses and areas

**Figure 7:**
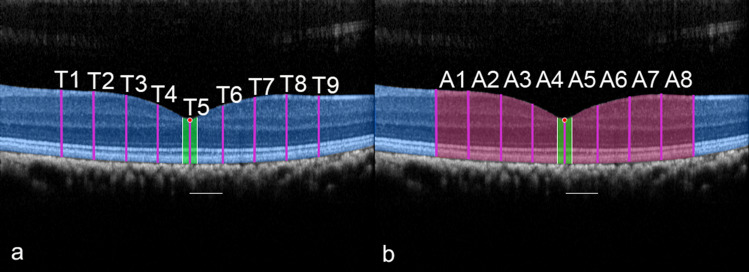
One- and two-dimensional retina parameters are illustrated in a left retina. From the anatomically deepest location within the foveolar cavity (marked as nulla, red dot) and orthogonally to the retinal pigment epithelium, side-by-side measurements were taken at 500 µm intervals starting nasally toward the temporal retina with respect to the axial retina diameters (**a)** (shown as pink lines and marked as retinal thickness parameters T1–T9) and the retinal surface areas (**b**) in between (highlighted in red and marked as areas A1–A8). Subfield analyses included the regions at a distance of 100 µm from nulla (highlighted with white lines and umbo surface area in green). The same procedure was performed for all eyes. For a better understanding, the segmented retina is depicted as a blue overlay. Scale bars, 500 µm.

The sixth algorithmic step is the measurement of retinal thicknesses and its areas. Using the semantic segmentation of the retina compartment on the central B-scan and the position of the anatomically and structured OCT-determined nulla, an imaginary line was orthogonally positioned in relation to the underlying retinal pigment epithelium to determine the axial diameter (Fig. 7). Subsequent measurements of retinal values were made at intervals of 500 µm to the side, up to a maximum of 2000 µm distance from the nulla. This allowed for measurements of nine retinal diameters (marked as thickness parameters T1–T9) in the axial direction, as well as eight intervening retinal areas (A1–A8), hence providing a total of 17 parameters to quantify the retina features (Fig. 7).

An additional subanalysis in the important area of the umbo was performed to determine the parameters for the most central receptors, which are responsible for the best visual acuity (central bouquet of cones)^41,51^. For this purpose, the thickness values and retina intervening surface areas were each determined laterally at an interval of 100 µm to the nulla. Thus, four further parameters were added: one additional nasal thickness (TUn), one temporal thickness (TUt), and two additional retinal surface areas from the nasal (AUn) and temporal (AUt) retina areas. Finally, including T5 (also indicated as the nulla), the umbo subfield analysis used a total of five parameters.

The first algorithmic step was performed in Python v3.5 and TensorFlow v1.14.0^69^. Steps two to six were performed in C# (v7.0, NET Framework v4.6). The combination of CNN-based semantic image segmentation with a fully automated fovea-finding algorithm, which is based on classical computer vision, can be described as “hybrid image processing.”

### Data analysis

#### Summary statistics and visualization

The summary statistics of the mean, minimum, and maximum were calculated for each of the measured retinal thickness and area coefficients on subsets of the data. Boxplots were used to visualize the distribution of the data and compare the two groups with each other (e.g., male vs. female). For the nulla, its retina diameter (T5), its adjacent retina surfaces (A4 and A5), and the average mean values were calculated for all eyes and both sexes.

#### Statistical analyses

Statistical analyses were performed (1) to investigate the observed variability in the data and (2) investigate differences with respect to sex and origin. The statistical analyses consisted of four parts.

First, a Pearson correlation analysis was performed to investigate the correlation among the 11 thickness and 10 area coefficients. As a result of the high correlation between (1) the thickness and area coefficients and between (2) the umbo subanalysis coefficients and T5, it was decided to include only the nine thickness coefficients T1–T9 in subsequent statistical analyses. For the Pearson correlation analysis, the left and right eyes were combined. Separate analyses of the left and right eyes yielded results almost identical to the results of the left and right eyes combined.

Second, a principal component analysis (PCA) was performed separately for the left and right eyes^70^. PCA does not provide insights into the effects of the independent variables of sex and origin but can identify the latent factors underlying the variability observed in T1–T9. Scree plots were used to visualize the eigenvalues of each PC. Each eigenvalue corresponds to the amount of variability explained by the corresponding PC. PCA coefficients are shown for the first three PC.

Third, a two-way MANOVA^71^ was used to jointly investigate the effects of the independent variables of sex and origin on T1–T9. MANOVA assumptions were checked with diagnostic plots (not shown). One multivariate outlier was removed from the group of right eyes. Wilks’ lambda was used to measure the impact of sex and origin. The MANOVA was separately calculated for the left and right eyes.

Fourth, two-way ANOVA tests were used to investigate the effects of sex and origin for each coefficient T1–T9 individually. The coefficients T1–T9 are obviously correlated with each other and, thus, not independent. Consequently, the ANOVA results are not independent of each other, and the *p*-values might be inaccurate. Nevertheless, we decided to perform individual ANOVA analyses because it allowed us to gain insights into which parts of the retina are responsible for the differences between male and female and Asian and Mauritius monkeys. To adjust for multiple testing, we performed Bonferroni corrections by dividing the significance levels by the number of tests, which was nine in our case. Variables that were significant at *p* < 0.001/9 are indicated with “***.” Variables that were significant at *p* < 0.01/9 are indicated with “**,” and variables that were significant at *p* < 0.05/9 are indicated with “*.” Finally, variables that were significant at *p* < 0.1/9 are indicated with “.”. ANOVA assumptions were checked with the diagnostic plots (not shown). ANOVA tests were performed separately for the left and right eyes.

The 374 eyes contained 16 eyes of an unknown origin, which were excluded from the MANOVA and ANOVA analyses. Some monkeys contributed a left eye and right eye. Thus, the left and right eyes are not independent of each other. Consequently, PCA, MANOVA, and ANOVA analyses were performed for the left and right eyes separately. All summary statistics, data visualizations, and statistical analyses were performed in Python v3.8.5. PCA and statistical tests were performed with the Python package statsmodels v0.12.1. Visualizations were generated using the Python package Matplotlib v3.3.2.

## Supplementary Information

Supplementary Information.

## Data Availability

The measurement datasets generated and analyzed during the current study are included in this manuscript and made available as Supplementary Data S1.
